# Postbiotics: Mapping the Trend

**DOI:** 10.3390/nu16183077

**Published:** 2024-09-12

**Authors:** Veroniki Stelmach, George Stavrou, Ioannis Theodorou, Eleni Semertzidou, Georgios Tzikos, Alexandra-Eleftheria Menni, Anne Shrewsbury, Aris Ioannidis, Katerina Kotzampassi

**Affiliations:** 1 Department of Surgery, Aristotle University of Thessaloniki, 54636 Thessaloniki, Greece; stelmachveroniki@gmail.com (V.S.); gtziko@auth.gr (G.T.); alexmenn@auth.gr (A.-E.M.); a_shrewsbury@yahoo.com (A.S.); ariioann@yahoo.gr (A.I.); 2 Department of Surgery, Addenbrooke’s Hospital, Cambridge CB2 2QQ, UK; stavgd@gmail.com; 3 Hellenic Institute for the Study of Sepsis (HISS), 11528 Athens, Greece; itheodorou@med.uoa.gr; 4 Library of AHEPA University Hospital, 54636 Thessaloniki, Greece; elenisemer@gmail.com

**Keywords:** postbiotics, microbiome, probiotics, bibliometry, review articles

## Abstract

Background: Since the consensus of ISAPP on the definition of the term “postbiotic” there has been an enthusiasm for publications in review form—their number being disproportionate to the primary research. The aim of this bibliometry is to analyze the bibliometric trends of this newfound interest in the field. Methods: Search of the PubMed database for review articles on postbiotics, published between November 2021 and June 2024. Results: Analysis was performed on 92 review articles, the number corresponding to 2.9 reviews per month. China, Poland, Italy, Iran and India had the maximum productivity among the 32 countries involved; 21 articles were published in 13 journals with the highest impact factor, while 45 were in 16 journals with an IF between 4.0 and 4.9. The authors were mainly affiliated to universities with specialization in both basic research and technology, as well as food science. The top five publications regarding the citations received, published in *Foods* (2), *EBioMedicine*, *Biomolecules*, and *Front. Nutr.*, have collected between 138 and 109 citations. Conclusions: The ever-growing number of reviews regarding postbiotics is perhaps disproportionate to the actual original research in the field. Further clinical trials would extend and deepen the subject and facilitate the drowning of more robust conclusions in relation to their effects.

## 1. Introduction

In the early period of experimental trials on the possible beneficial effects of probiotics and their action pathways, it was a fairly common practice to use a “heat-killed probiotic” group as a kind of second control group [1,2,3]. This group was used to address the question of whether living or dead bacteria or any of their organelles or bacteria components are actually beneficial. With time, it became apparent that, practically, there was no difference in how the probiotics work and so, slowly, the practice of using a heat-killed probiotic group ceased.

However, the enormous progress achieved recently in the use of cell cultures for specifically testing the immunomodulatory effects of probiotics on a distinct cell type, i.e., keratinocytes or endothelial cells, has revived the old practice of using dead bacteria, now in the form of bacteria lysates—independently of the method used for their lysis—in order to make the experimentation easier and less interrelated to external factors and/or the peculiarities of the organism as a whole [4,5].

The same subject, viewed from another angle, that of the clinician, has brought confirmation of the beneficial effect of non-viable bacteria, their fragments, or by-products and, in the next few years, will resolve the presumed risk of bacteremia induction in the severely ill, immune-compromised patients treated with probiotics [6].

However, this new era of knowledge has also led to a variety of terms used to refer to them, such as heat-killed, inactivated or non-viable, dead probiotics, paraprobiotics, postbiotics, cell fragments, and bacterial lysates that all describe the same entity but unfortunately result in confusion with the terminology [7]. Thus, in October 2021, a group of experienced scientists, from both basic science and clinical fields, under the auspices of the International Scientific Association of Probiotics and Prebiotics [ISAPP] convened to clearly determine the conditions under which the various “modified forms” derived from live probiotics could be referenced under the same name: “postbiotics”. Today, postbiotics are defined as the “preparation of inanimate microorganisms and/or their cellular components that confers a health benefit on the host”, including in this the killed microbial cells, with or without metabolites and excluding purified products, i.e., proteins, peptides, exopolysaccharides, and Short Chain Fatty Acids [8].

The establishment of this consensus led to a surge in publications both experimental and clinical, as well as in review form, with indeed, the reviews being numerically disproportionate to the “production” of primary research, experimental or clinical. The present study, therefore, aims to provide insights and analyze the review articles referring to postbiotics, published after October 2021, in order to map the trend of publications on this new topic.

## 2. Materials and Methods

### 2.1. Search Strategies

The literature search was performed between 10 and 14 July 2024, the required data were retrieved from the Pub-Med database, by using the unique term “postbiotics”. Then, filters were applied: we selected only those publications characterized by the system as “review” and “systematic review”, and then applied a time frame from 1 November 2021 to 30 June 2024. The month of October 2021 signaled the beginning of publications on “postbiotics”, following the adoption of the term by the consensus paper published that month [8]. No language restriction was applied.

The articles found were thoroughly assessed by two authors, KK and GS, who independently initially screened the titles and abstracts. In the case of discrepancy, the issue was resolved by reading the full text and then screened by a third, totally independent reviewer [EE]. For further processing, the selected articles were then downloaded, put into a comma-separated values format, and imported into Microsoft Excel 2019.

### 2.2. Initial Data Retrieved from the Selected Articles

The two authors who initially reviewed the titles then went through the full texts of all selected publications to retrieve the following data: [i] demographics: publication year, names of all authors, names of the first and last author of each article, gender of the first author, journal name, and journal impact factor; [ii] statistics: the total number of authors cumulatively, the number of first only, and last only authors; [iii] duplications: the number of authors of each category, i.e., total, first, and last authors, who had authored more than one paper; [iv] affiliations of first and last authors: country/countries involved, country collaboration and stratification of their institutions into categories: universities [other than medical ones], medical universities, hospitals [non-affiliated to universities], research institutes non-affiliated to universities, and industry; [v] the field of interest of first and last author’s work, i.e., genetics, biotechnology, dairy products, food, skin/cosmetics, internal medicine and so on.

Statistical assessment of data was not generally necessary for the present study, except for a median value calculation. All figures and tables were created using Microsoft Excel 2019, on a MacBook, except Figure 7, which has been drawn in Microsoft Power-Point 2019, [16.7] for MacBook. Whenever information had to be ranked, the top 10 components were reported, unless otherwise stated.

## 3. Results

**Figure 1:**
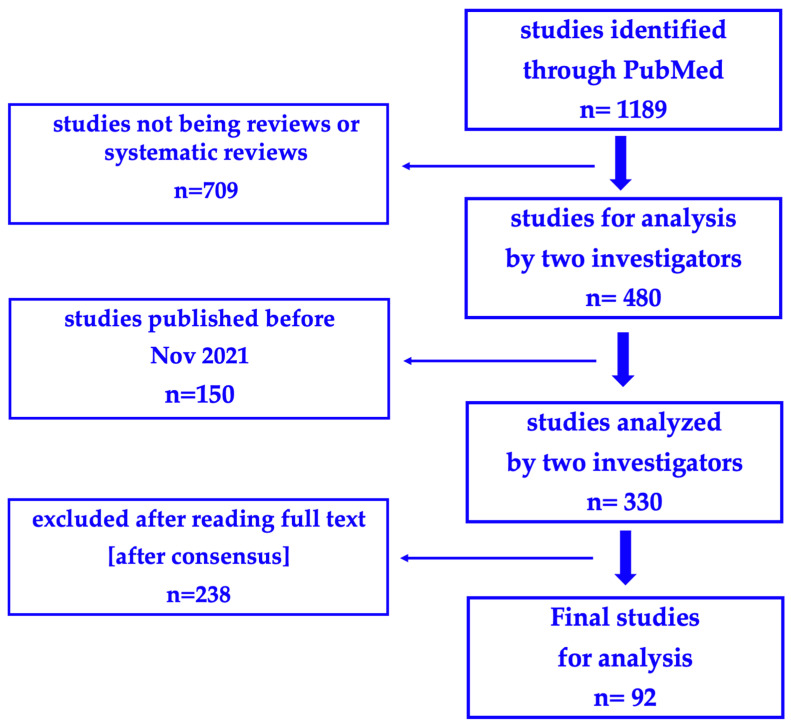
Flowchart.

Our search strategy in the PubMed database initially identified a total of 1189 records. After setting as screening filters the terms “review” or “systematic review”, 709 publications were excluded; following the next filter, relating to the publication time frame [1 November 2021 to 30 June 2024], another 150 were excluded, leaving 330 for analysis. These studies were initially screened by two authors, independently, and in the case of discrepancy, by a third. Finally, 92 articles remained to be analyzed after the exclusion of a further 238. The search outcome is displayed as a flowchart in Figure 1.

### 3.1. Initial Data Retrieved from the Selected Articles

#### 3.1.1. Demographics

**Figure 2:**
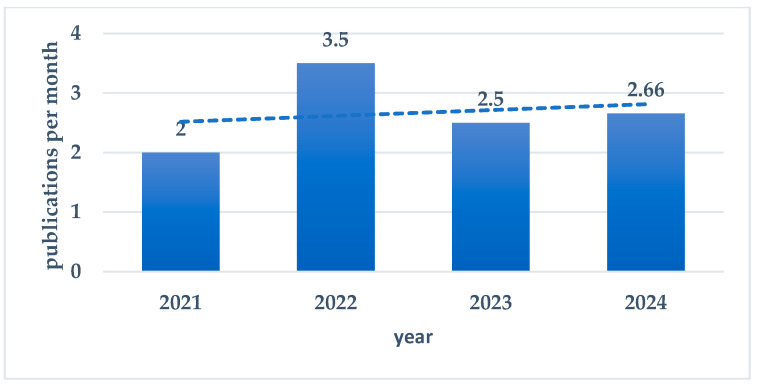
Monthly publication rate per year.

The search covered a 32-month period, beginning in November 2021, the date of the publication of the consensus formally defining the term “postbiotics” [8]. In total, 92 review articles were found to be eligible for analysis; four were published in 2021, 42 in 2022, 30 in 2023, and 16 in 2024 [up to the end of June]. The monthly publication rate per year is presented in Figure 2.

The cumulative demographic characteristics retrieved from the 92 review articles under analysis are presented in Table 1.

**Figure 3:**
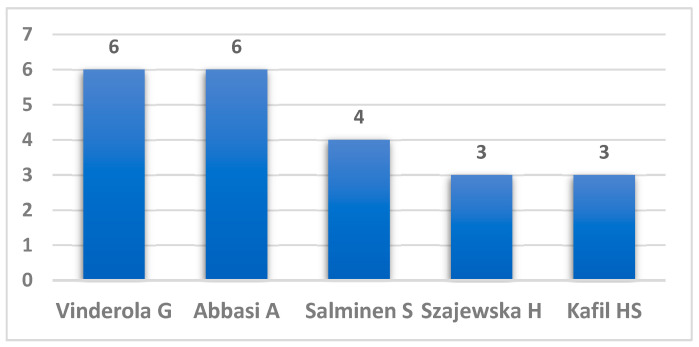
Authors contributing to more than two publications.

The 92 papers have a total of 488 authors. Of these—regardless of the authorship rank—Vinderola G, from Argentina, and Abbasi A, from Iran authored six papers each—which means their names were found six times within the 488 authors—Salminen S, from Finland, four, and Szajewska H from Poland, and Kafil H from Iran, three papers each (Figure 3). Additionally, another 21 scientists authored two papers each. This means that 456 scientists authored in collaboration with others, one or more of the 92 articles.

**Figure 4:**
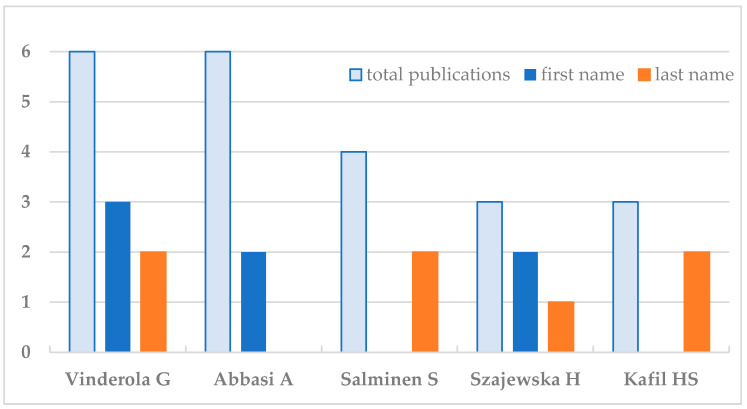
Scientists contributing with more than one publication, stratified as first and/or last authors.

From the 26 authors with more than one publication, most were either first or last authors: Vinderola G, having authored six papers, was first author in three and last author in two; Abbasi A having authored six papers was first in two; Salminen S with four publications and Kafil HS with three had authored two papers each as seniors; and Szajewska H having authored three papers was first author in two and senior in one. From the nine authors of two papers, Ozma MA and Martyniak A were first author in two and one, respectively; Wędrychowicz A, Tomasik PJ, Shahbazi N, and Rahbar Saadat Y, had one publication each as last/senior author; and three had no papers as first or last author (Figure 4).

Given that two out of the 92 papers had a single author, we have 92 first and 90 last authors. Or, after taking into consideration the multiple authorship of some scientists, we finally had 87 first and 85 last authors.

After the exclusion of the 92 first and the 90 last authors, there remained 306 authors serving as authors in the middle ranks, meaning there is a median of three [IQR: 2] middle authors in each paper, ranging from 0 to 18. These authors are outside the parameters of this study and will not be further referred to.

#### 3.1.2. Countries of Affiliation of First Authors

**Figure 5:**
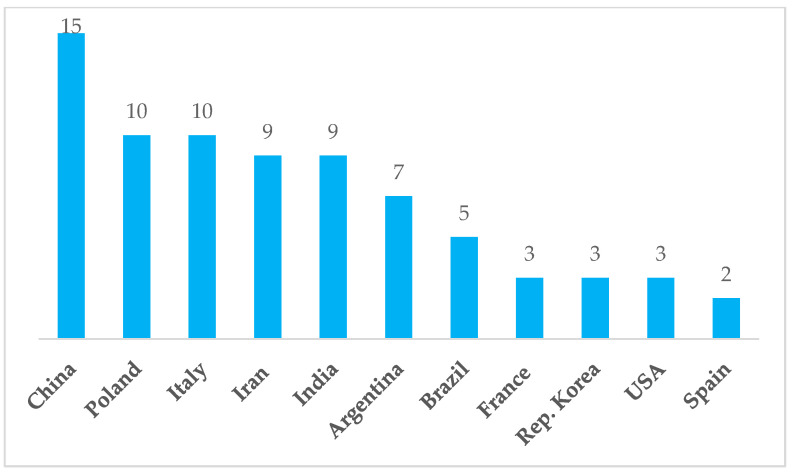
Eleven countries of first authors, having at least two publications each.

According to the country of residence and work of the first authors, eleven countries had the greatest productivity—76 articles—contributing two or more papers each; thus, 76 publications came from 11 countries and the remaining 16 were from another 16 countries. China was top with 15 publications, followed by Italy and Poland with 10, and India and Iran with 9 (Figure 5).

Four authors presented having double-country affiliation, but only the first mentioned was counted: Spivak I. comes from Israel and Germany; Scarpellini E. from Italy and Belgium; Vera-Santander VE. from Mexico and UK; and Bozzetti V. from the USA and Italy.

#### 3.1.3. Countries of Affiliation of Last Authors

**Figure 6:**
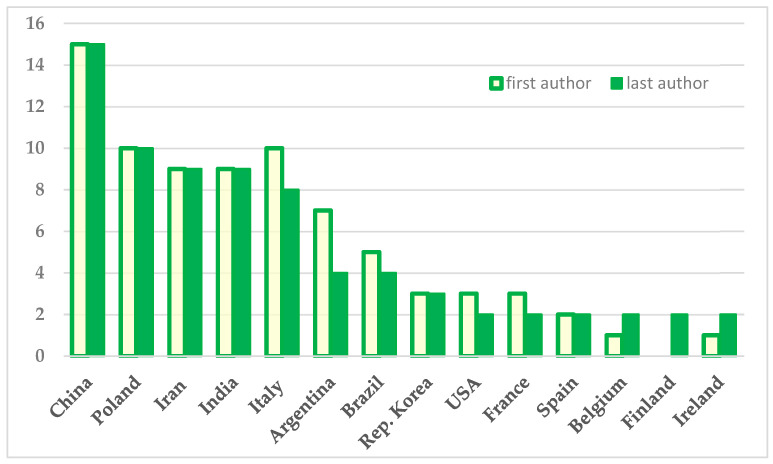
Number of articles per country, according to affiliation of the first and last authors, presenting only countries having a minimum of two publications.

The last authors came from 32 countries, fourteen of which had the greatest productivity—74 articles—contributing two or more papers each, and the remaining 18 publications came from another 18 countries. As a consequence of the collaboration among countries, there is a slight difference in the rank of countries from which the last authors came in relation to that of the first authors. The country with the highest productivity was China again, with 15 publications [rate 16.3%], followed by Poland with 10 [rate 10.9%], India and Iran with 9 [rate 9.8%] each, Italy with 8 [rate 8.7%], and Argentina and Brazil with 4 each [rate 4.3%]. The number of studies per country is presented in Figure 6 along with the first and last authors for each country.

A significant difference was observed in relation to Argentina, which, while having seven articles of first authorship, had only four in the list of last authors, the difference being attributable to its collaboration with other countries. A similar result is observed with Brazil, France, and the USA—one publication less than the number with first authors. The opposite is true for Belgium and Ireland which have two articles in the last but only one in the first author list. Finally, Finland, Bangladesh, Colombia, Canada, and Thailand had no publications from first authors, but did have last authors—Finland had two and the other four had one each.

#### 3.1.4. Country Collaboration Networks

In sixty-five out of the 92 articles [rate 70.6%], all the authors came from the same country, while the remaining 27 articles were the result of collaboration between authors from two to seven countries: in 16 cases from two countries; in six from three; and five publications coming from the collaboration of four, four, five, six and seven countries. The seven-country collaboration came from France [with Mexico, Singapore, Italy, Belgium, Vietnam, and Ireland]; the six-country collaboration came from India [with UAE, USA, Australia, Chile, and Brazil]; and the five-country collaboration from Argentina [with France, Spain, Finland, and Belgium] and from India [with Brazil, Australia, Chile, UAE and the USA]. Finally, the four-country collaborations came from Argentina [with the USA, Finland, and Poland] and Iran [with the USA, Denmark, and Azerbaijan].

**Figure 7:**
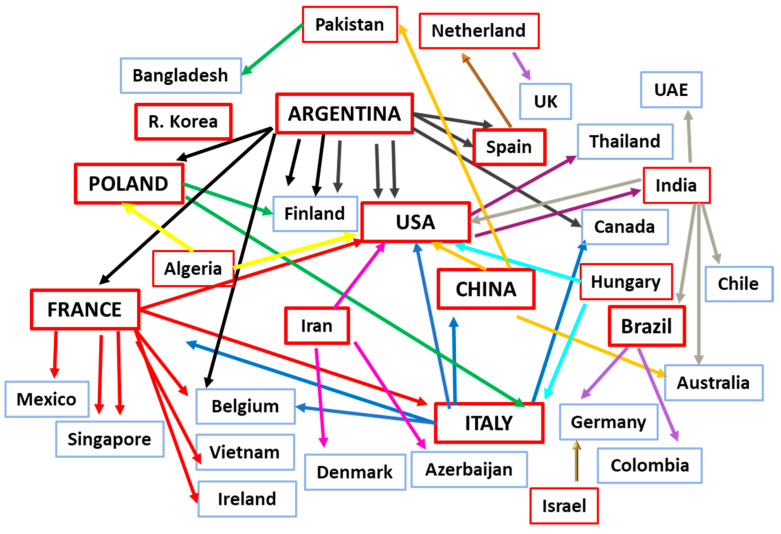
Country collaboration [one arrow represents one paper]: red frame for active countries; the direction of arrows indicates the cooperative country [blue frame].

It is of interest to note that of Argentina’s total of seven publications, five involved collaborations with other countries, meaning co-authorship with a total of 11 countries. France had collaborations with eight countries. Poland’s total of 10 articles, however, involved collaboration with another country in only two of them [with Italy and Finland]. China, with a total of 15 articles, collaborated with other countries in only three instances [USA, Australia, or Pakistan]; and Iran with nine articles collaborated in only one case—with the USA, Denmark, and Azerbaijan (Figure 7).

### 3.2. Institutions of First and Last Authors

**Figure 8:**
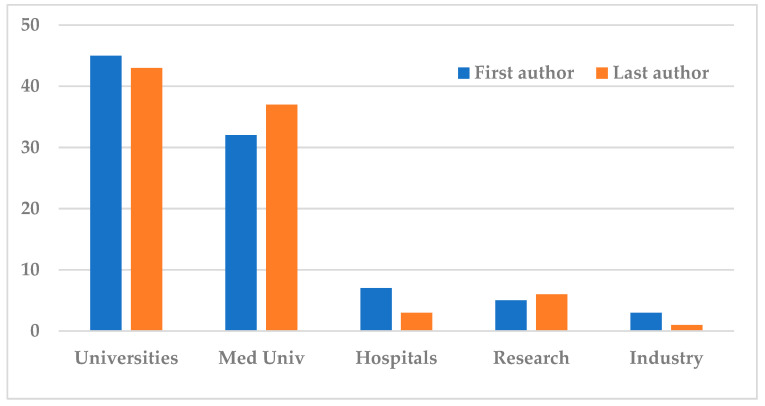
Affiliations of first and last authors.

The first authors of the 92 publications were affiliated with universities [n = 45], medical universities [n = 32], hospitals [not related to medical schools, n = 7], independent research centers [n = 5], and industry [n = 3] (Figure 8).

The last authors of the 90 publications [two articles out of the 92 had a single author counted as first] were affiliated with universities [n = 43], medical universities [n = 37], hospitals [not related to medical schools, n = 3], independent research centers [n = 6] and industry [n = 1]. Three authors—Viderola G, Salminen S, and Kafil HS—authored two articles each as seniors, but counted as different authors [Figure 8].

### 3.3. Specialties of First and Last Authors

**Figure 9:**
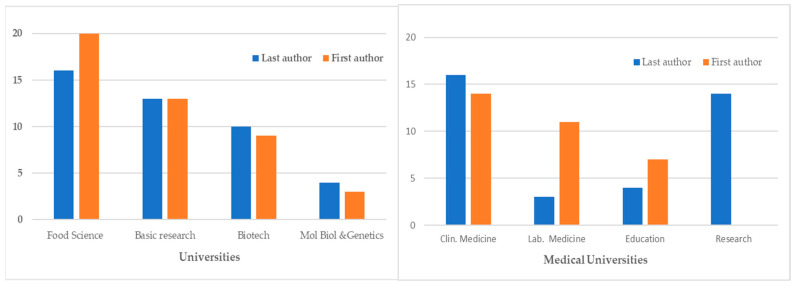
Specialization of first and last authors affiliated with universities.

Regarding first authors, the 45 articles that came from non-medical universities were from departments dealing with food science [n = 20, including five in dairy science], basic research [n = 13], biotechnology [n = 9], and molecular biology and genetics [n = 3]. The 32 papers from medical universities were from clinical [n = 14] and laboratory [n = 11] medicine departments, as well as another seven from student research. The seven articles from non-university hospitals were as follows: four from the departments of pediatrics and one each from departments of internal medicine, nephrology and hypertension, and arthritis. Finally, five articles from research laboratories—not affiliated with universities—were from biochemistry, biotechnology, immunology, and food science departments, while three further articles came from industry and are related to research in dermatology [n = 2] and nutrition [n = 1] (Figure 9, Table 2).

Regarding last authors, the 43 articles that came from non-medical universities were from departments dealing with food science [n = 16, including one in Dairy Science], basic research [n = 13], biotechnology [n = 10], and molecular biology and genetics [n = 4]. The 37 papers from medical universities were from clinical [n = 16] and laboratory medicine [n = 3], from research [n = 14], as well as another four from student research. The three articles from non-university hospitals came from departments of pediatrics, emergency medicine, and geriatrics. Finally, six articles from research laboratories—not affiliated with universities—were from biochemistry [n = 2], biotechnology [n = 1], immunology [n = 1], and food science [n = 2] departments, while one article, that came from industry, was related to research in dermatology (Figure 9, Table 2).

### 3.4. Journals of Publication

**Figure 10:**
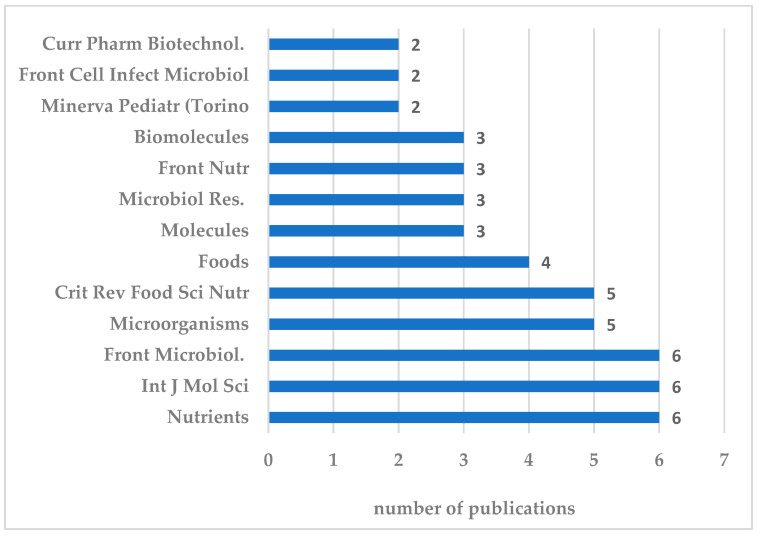
Thirteen journals with two or more publications.

The 92 articles were published in 51 journals: thirteen journals hosting more than half of the publications, [n = 50, rate 54.3%]. *Int. J. Mol. Sci.*, *Nutrients*, and *Front. Microbiol.* were ranked first in frequency, with six publications each; followed by *Crit. Rev. Food Sci. Nutr.* and *Microorganisms*, with five publications each (Figure 10).

### 3.5. Journal Impact Factor

**Figure 11:**
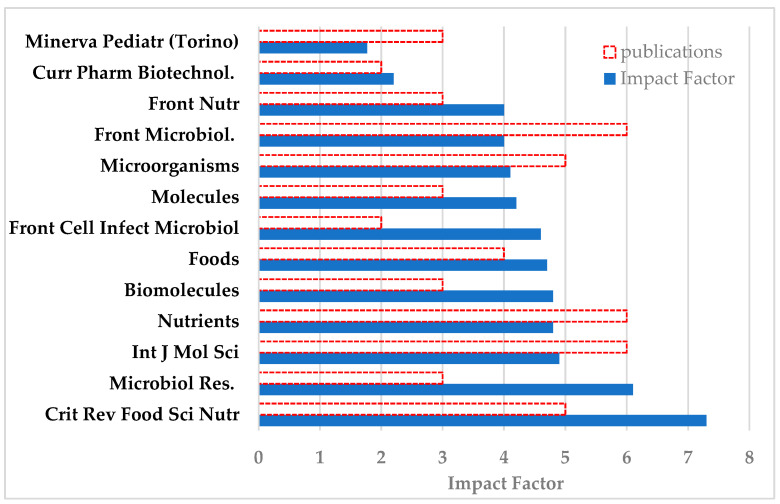
Thirteen journals with two or more publications [red open bars] and their Impact Factors [blue bars].

Among the 13 journals hosting the 50 articles, *Crit. Rev. Food Sci. Nutr.*, with five publications, has the highest IF of 7.3, followed by *Microbiol. Res.* with three publications and an IF of 6.1, next are *Int. J. Mol. Sci.* with 4.9 and *Nutrients* with an IF of 4.8—six publications each (Figure 11). All IFs were correct as of August 2023.

Six out of the 13 journals belong to the publisher MDPI, three to Frontiers, one to Elsevier, and one each to Taylor & Francis, Bentham, and Minerva Medica publishers; this means that 27 out of 50 articles are published by MDPI journals [rate 54%], 11 by Frontiers [rate 22%], five by Taylor & Francis, three by Elsevier, two by Bentham, and three by Minerva Medica. However, the remaining 42 articles are also published in significant journals, some of them with a higher impact factor than those given above.

**Figure 12:**
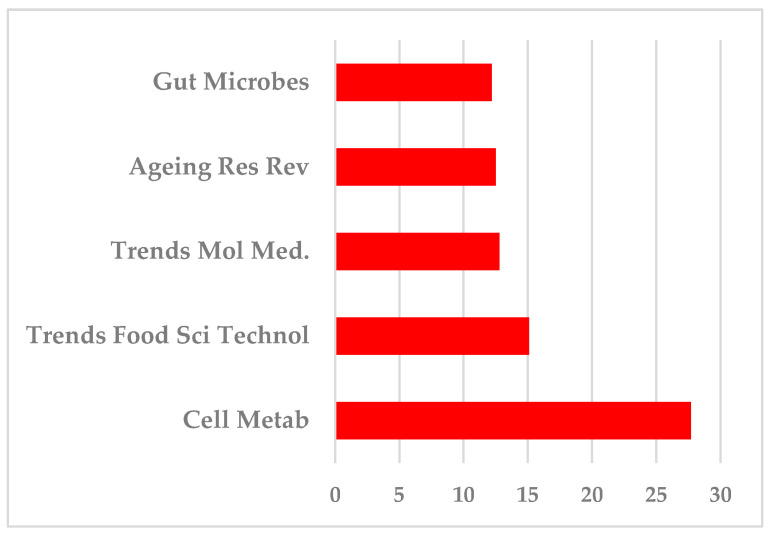
Top five journals among the 92 analyzed, according to the Impact Factor.

Regarding the total of 92 publications analyzed, five of them [rate 5.43%] have been published in journals with an Impact Factor of between 27.7 and 12.2 (Figure 12).

**Figure 13:**
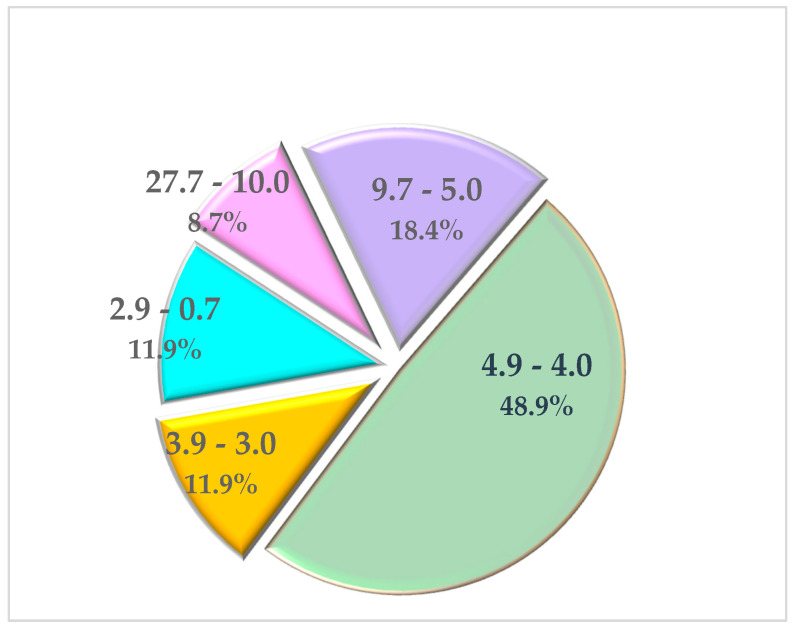
Distribution of journals based on their Impact Factor and the articles hosted.

When we classified all journals hosting the 92 publications according to their Impact Factor, we found six journals with an Impact Factor of between 27.7 and 10.6 hosted 8 publications; 11 journals of between 9.7 and 5.0 hosted 17 publications; 16 journals of between 4.9 and 4.0 hosted 45 publications; 9 journals of between 3.9 and 3.0 and the remaining 9 journals of between 2.9 and 0.7 hosted 11 publications each. It is of interest to mention that almost half the articles [n = 45] are published in journals with an IF of 4.9 to 4.0 (Figure 13).

### 3.6. Number of Citations Received and Most Cited Authors

Because of the limited time frame of analysis [November 2021 to June 2024], there has only been a short time for the articles to be cited. At that moment [16 August 2024] seven articles have not yet received any citations, 32 articles have received a median of three citations [IQR 2, 7], and another 43 have a median of 20 [IQR 15, 31]. All data were obtained from Google Scholar. Finally, the top 10 publications with the highest number of citations are presented in Table 3.

At the top, with 138 citations, is the paper by Vinterola G from Argentina, which was published in April 2022. It is a review article, explaining in some way the cornerstone article by Salminen S, et al. [8] that established the term “postbiotics”. Unexpectedly, the article by Scarpellini E, et al. from Italy in collaboration with Belgium, published in *Int. J. Environ. Res. Public Health* [IF, 4.6], although published earlier—December 2021—has only received 54 citations, thus classified in the 9th rank. However, Martyniak A, from Poland, having the same advantage of earlier publication—December 2021—has received 119 citations [3rd rank], probably due to the hot topic of IBD it is dealing with.

Looking into these top 10 authors/articles we note that from the authors with more than one publication as first or last, Vinderola G. and Abbasi A. have the 1st [138 citations] and the 7th rank [82 citations]. Finally, it is of interest that two papers published in April and March 2023, take the 2nd and the 10th rank; both are based on clinical research [depression and oral health] which means that they have a multifaceted audience.

## 4. Discussion

Bibliometric analysis is an effective numerical analysis measure of the scientific contribution to a specific research field in a specific period of time, as well as of the relationships between these publications, i.e., to ascertain which authors, scientific centers, countries, and journals have the greatest influence in the advances in the given field of science [9]. Bibliometry represents a branch of the library and information sciences that, by using statistical methods, analyzes research articles, both to obtain a general idea of the current state of research on a narrow scientific topic of interest and to measure the scientific level of the researchers/authors of the relative publications [10,11].

To our knowledge, this is the first bibliometric analysis that presents the dynamics of the production of a specific category article in this field. We analyzed publications on postbiotics with the peculiarity of only selecting review articles authored within a very strict time frame, ultimately aiming to map the trend on the emerging concept of postbiotics. A review article is an attempt by one or more scientists to perform a comprehensive summary of the current understanding of a specific research topic, or a critical analysis of current thought in the field, based on previously published research, and, more importantly, to focus on discrepancies in the literature, gaps in knowledge, and recommendations for future research, concluding with comments inspired by their own knowledge and experience. In other words, a review is considered a critical analysis of current thought in the field, the basis for this, and the strength of the evidence, followed by suggestions for new directions of research. Thus, a review is generally written by a person—and his/her colleagues—who has gained some authority on the particular topic after working for a number of years in the given field and with relevant research publications of their own [12,13,14].

In this present bibliometric analysis of 92 review papers dealing with postbiotics, we found 488 authors, since each review had between one and 18 authors. These authors, for technical reasons, were studied after having been separated into first and last authors, and those of the middle ranks. The 306 middle-rank authors are mainly young in age, new investigators beginning their career right now in a research laboratory of a university or an institution. On the other hand, they may be co-researchers in a large project or even senior researchers in a multi-department or multi-country publication. And, as we have found, 27 articles were the result of collaboration between authors from two to seven countries: in 16 cases from two countries; in six from three; and in five publications coming from the collaboration of four, four, five, six, and seven countries. This multiplicity also explains the large number—up to 18—authors in some studies. Our decision to take into account the first and last authors, separately, was based on the assumption that the first name is generally the main author responsible for the paper, and the last is either the senior author or possibly the supervisor of the work [13].

Therefore, we have an author from Argentina, [Vinderola G] who serves as first author in three articles, as last author in two, and as a middle author in another one multicentric publication. Similarly, Abbasi A, from Iran, has also authored six papers, in two of which he is the first author. The same is observed with Salminen S, from Finland, who served as last author in two and as a middle author in another two multicentric publications, as well as Szajewska H, from Poland, who was first author in two, and middle and last author once in each position. Finally, Kafil H from Iran, with three papers was first author in two and middle in one. Additionally, another 21 scientists authored two papers each. Conclusively, all these authors seem to have huge experience in teaching and serving as mentors to their young colleagues, and this comes from their involvement in the relevant topics of probiotics and microbiome. For example, we have found that among the previously mentioned multi-article authors, Salminen S had a total of 142 publications relating to probiotics and/or microbiome, Szajewska H, 92, and Vinderola G, 41. It is also important to note that the great majority of these authors who have a double-digit number of publications on the microbiome and/or probiotics are also positively involved as members of the board of directors in the consensus statement on the definition and scope of postbiotics organized by the International Scientific Association of Probiotics and Prebiotics [ISAPP].

A remarkable point of our analysis is the countries of affiliation of first and last authors as well as the degree of collaboration among countries. Not unexpectedly, China was the first in the number of publications, having 15; this was in some way expected because of its huge population and the thousands of universities and affiliated research institutes. Among these 15 publications, only three were written in collaboration. However, all of France’s three articles were written in collaboration with other countries; the top being the unique seven-country collaboration [with Mexico, Singapore, Italy, Belgium, Vietnam, and Ireland]. The situation in Argentina was similar; five of their seven publications were in collaboration with other countries, thus achieving a co-authorship with a total of 11 countries. It is easily understandable that these collaborations are directly related to the obstacle-free movement of scientists, mainly towards Europe and less to the USA, but also to the strong personal contacts of some researchers with others, worldwide, not to forget the major role of ISAPP in this bond.

Another observation of our bibliometry is related to the institutions and the specialization of the centers of origin of the publications. Regarding the first authors [n = 92] 45 were affiliated with universities, and 32 with medical universities, while far fewer were those affiliated with hospitals not related to medical schools [n = 7], independent research centers [n = 5], and industry [n = 3]. The data for the last authors was similar, that is 43 were from universities and 37 from medical universities, while three, six, and one were from hospitals, research centers, and industry, respectively. These numbers clearly reveal the low rate of clinical research in medicine. Of course, we acknowledge the difficulties and restrictions to performing research studies involving humans, which are even greater in relation to sick patients. That is not to say that research involving animals or cell cultures is easier or simpler, but, despite basic research being acknowledged as both invaluable and irreplaceable, we urgently need applied medical research, both to improve health and, primarily, to treat disease. It is nonetheless true that, from a practical point of view, medical doctors in clinical practice lack the same opportunities and time to write scientific work in comparison to full-time researchers [15]. Today, postbiotics are now widely recognized, due to their benefit of not being live microbes, to be the opening of new horizons of hope for thousands of immune-compromised patients hesitating to receive probiotics or whose physicians are reluctant to prescribe them [6,16].

The most known and used bibliometric measure is the journal impact factor [IF]. In recent years it is acknowledged to be the basic measure of the quality of scientific research output, although it has its limitations. Authors in general, as a quantifiable measure of their scientific success [17], aim to publish in the most known journals, which are the most read and consequently most referenced, which finally means they have the highest impact factor, although this measure clearly reflects the journal’s scientific impact and not that of the individual article. On the other hand, grant agencies take into serious account the journals in which the applicant has published to rate their track record [13], while the open access policy highly increases the visibility of the articles. In the present bibliometry we found 50 articles that have been published in only 13 journals, having an impact factor from 7.3 [*Crit. Rev. Food Sci. Nutr.*, five articles] to 2.2 [*Curr. Pharm. Biotechnol.*, two publications]. This finding indicates that, besides the desired high Impact Factor, there are some journals more preferred by authors, due to either their high specificity or the relatively high speed of the process of peer review/acceptance and publication, or both. On the other hand, there are five articles [rate 5.43%] published in journals with a high Impact Factor of between 27.7 and 12.2, while 45 publications are hosted in 16 journals with an Impact Factor of between 4.9 and 4.0. Among these most “popular” journals [11] are the *Int. J. Mol. Sci.* [IF 4.9, six publications], *Nutrients* [IF 4.8, six publications], *Microorganisms* [IF 4.1, five publications], and *Front Microbiol* [IF 4.0, six publications].

Nonetheless, it is not entirely correct to assess the scientific impact of a publication and compare it with another in terms of the Impact Factor of the journal of publication, since a journal’s Impact Factor depends on many things, including the publication area of the journal and the nature of the research [basic research or clinical practice] [17].

On the other hand, it is well known that the number of times an article is cited is a good way of measuring its impact in a specific field over a period of time, and this in turn allows the evaluation of both the authors and the journal itself [18]. However, it is important to understand that gathering citations is a time-dependent process, a new study, in whatever category, requiring at least two to five years for accumulation of enough citations to be used as a reliable bibliometric indicator [19]. In the present analysis, the time frame—November 2021 to June 2024—is seriously prohibitive; surprisingly, two papers published in early 2023 keep the 2nd and the 10th rank, an obvious explanation could be that these two articles are based on clinical research [depression and oral health], making them readable and citable by researchers from different specificities.

It is believed that an article by an author well-known in his/her scientific field will have many citations. The same is considered true for publications resulting from an international collaboration [20]. In the present list of the top 10 most cited articles only the publication by Vinderola, et al., in the 1st rank with 138 citations, fulfills both characteristics: It is authored by three well-known scientists—Vinderola G, Sanders ME, and Salminen S and comes from the collaboration of three countries: Argentina, the USA, and Finland.

The present bibliometry does have some potential limitations: [i] only the PubMed database was used; however, it is a commonly used and reliable source for bibliometry in the fields of medicine and biomedical sciences [21]; [ii] we distributed collaboration equally among countries, although the degree of involvement of each author was not known. Sometimes, a scientist contributes as an author while a visitor to a Lab, but his country is written as his affiliation; [iii] unfortunately, it was not possible to assess the quality and impact of the publications by the citations list, as almost all the articles published in 2023 have very few or even zero citations; the same also applies for the h-index; [iv] it is also well understood that it was not possible by means of bibliometry to assess the scientific soundness of every review study; and, it is not automatically self-evident that every review written by an established scientific author is of top quality; and [v] the study design excludes analysis of all the middle-rank authors. This resulted in the “loss” of these authors from at least 27 articles written in collaboration between different countries/universities.

## 5. Conclusions—Final Comments

To summarize: there is an ever-increasing interest, as demonstrated by the number of review articles, in the concept of “probiotics”. However, a mean of three review articles per month is somewhat excessive—what new can there be to say every ten days? Of course, these are published in different journals, probably addressing different audiences, but when searching a database for a certain topic we search for all aspects of it, which will present everything written. Assessing the reviews independently of their authors’ specific knowledge, specialization, scientific experience, and general productivity we recognized that probiotics/postbiotics are experiencing a consistent and ongoing, steady publishing trend on this topic. However, despite the pluralistic orientation of research centers, i.e., biochemistry, biotechnology, genetics, food chemistry, dairy products, and cosmetics, the involvement of medical doctors in applied [clinical] research is still in the early stages. This should be the final focus of all research efforts: not only to improve health but mainly to support and, even better, to treat the sick human patient.

## Figures and Tables

**Table 1:** General characteristics of the study.

	Number
Articles	76
Time frame for publication	November 2021–June 2024
Authors [authors by name]	488 [456]
First authors [by name]	92 [87]
Last authors [by name]	90 [85]
Authors at middle rank [median]	306 [3, IQR 2]
Countries of origin of first author	27
Countries of origin of last author	32
Total countries involved	50
Articles from international collaboration	27
Journals	51

**Table 2:** Specialization of first and last authors not affiliated with universities.

		First Author	Last Author
**Hospitals**	Pediatrics	4	1
	Internal Medicine	3	0
	Emergency Medicine	0	1
	Geriatrics	0	1
**Research**	Biochemistry	2	2
	Biotechnology	1	1
	Immunology	1	1
	Food Science	1	2
**Industry**	Dermatology	2	1
	Nutrition	1	0

**Table 3:** Top 10 publications, according to the number of citations received.

First Author	Title	Journal	Country	Journal IF	No of Citations
Vinderola G.	The Concept of Postbiotics.	*Foods*. 2022 April	Argentina	4.7	138
Liu L.	Gut microbiota and its metabolites in depression: from pathogenesis to treatment.]	*EBioMedicine*. 2023 April	China	9.7	119
Martyniak A.	Prebiotics, Probiotics, Synbiotics, Paraprobiotics and Postbiotic Compounds in IBD.	*Biomolecules*. 2021 December	Poland	4.8	119
Thorakkattu P.	Postbiotics: Current Trends in Food and Pharmaceutical Industry.	*Foods*. 2022 October	USA–India–Thailand	4.7	117
Liu Y.	Modulation of Gut Microbiota and Immune System by Probiotics, Pre-biotics, and Postbiotics.	*Front. Nutr.* 2022 January	China	4.0	109
Xavier-Santos D	Evidences and perspectives of the use of probiotics, prebiotics, synbiotics, and postbiotics as adjuvants for prevention and treatment of COVID-19: A bibliometric analysis and systematic review.	*Trends Food* *Sci. Technol.* 2022 February	Brazil	15.1	99
Abbasi A	The biological activities of postbiotics in gastrointestinal disorders.	*Crit. Rev. Food* *Sci. Nutr.* 2022	Iran	7.3	82
Bourebaba Y	Postbiotics as potential new therapeutic agents for metabolic disorders management.	*Biomed Pharmacother.* 2022 September	Algeria–Poland–USA	6.9	72
Scarpellini E	From Pre- and Probiotics to Post-Biotics: A Narrative Review.	*Int. J. Environ. Res. Public Health*. 2021 December	Italy–Belgium	4.6	54
Homayouni Rad A	A comprehensive review of the application of probiotics and postbiotics in oral health.	*Front. Cell. Infect. Microbiol.* 2023 March	Iran	4.6	51

## Data Availability

This study did not report any data.
